# Factors Influencing the Stability of Blood Eosinophils Counts in Chronic Obstructive Pulmonary Disease Patients

**DOI:** 10.1155/2022/8369521

**Published:** 2022-03-27

**Authors:** Cheng-Sen Cai, Jun Wang

**Affiliations:** Department of Respiratory and Critical Care Medicine, Second Affiliated Hospital of Shandong University of Traditional Chinese Medicine, Jinan, ShanDong 250001, China

## Abstract

Blood eosinophil (EOS) has recently been recognized as a biomarker for chronic obstructive pulmonary disease (COPD) patients. However, few studies have concentrated on the stability of blood eosinophil counts (BEC), and those studies have produced varying results. With further research, we have found minor drawbacks and vulnerabilities that lead to the variability of the results. This paper enumerates several areas of relevant research with varying conclusions to further investigate the stability of BEC in COPD patients.

## 1. Introduction

COPD is a common, preventable, and treatable disease that is characterized by persistent respiratory symptoms and airflow limitations caused by airway and/or alveolar abnormalities [1]. Blood EOS, a new biomarker, has been proposed as a guide that indicates clinical outcomes in COPD patients. The Global Initiative for Obstructive Lung Disease (GOLD) guidelines state that blood EOS levels are associated with inhaled corticosteroid (ICS) responsiveness, risk of pneumonia, and further exacerbations. Meanwhile, the GOLD devised different recommendations for ICS use to lessen the inappropriate use of steroids [1]. Many studies have focused on the threshold of the EOS to ensure the appropriate use of ICS. However, few studies have focused on the stability of BEC in predicting COPD outcomes. Surprisingly, the results on the stability of BEC were not consistent across studies. By focusing on those relevant studies, we find that the varying results are just a fraction of the inadequacy of current research in the area. Other minor drawbacks and vulnerabilities might have caused the varying results. Therefore, we have reviewed these studies to further investigate the stability of BEC.

## 2. Inclusion Criteria

**Figure 1:**
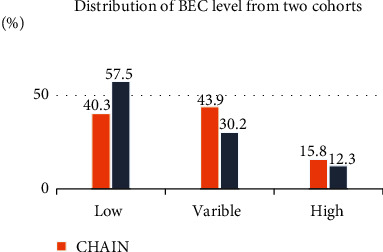
Distribution of BEC level from two cohorts. CHAIN: history of asthma was not excluded. BODE: history of asthma was excluded; cutoff value: 300 cells/*μ*L.

Inclusion criteria in each study matter a great deal. The diagnosis of COPD requires the presence of a forced expiratory volume in the first second/forced vital capacity (FEV1/FVC) that is <0.70. However, Shantakumar et al. often used specialists to diagnose COPD because patients were referred by their general practitioners [2]. Diagnoses that do not use spirometry risk confuse COPD with other diseases, such as asthma, and could therefore influence the results of any study. As is known, BEC can be abnormal in asthmatic patients. Subjects with a known history of asthma were excluded in some studies while studying the stability of BEC [3, 4]. However, results may have been affected if a prior history of asthma was not excluded. Casanova et al. assessed two large cohorts including the CHAIN (COPD History Assessment In Spain) and BODE (body mass index (*B*), degree of airflow obstruction (*O*), functional dyspnoea (*D*), and exercise capacity (*E*)) to study the stability of BEC [5]. Among them, asthma history was excluded in the BODE cohort but the CHAIN was not (Figure 1).

In addition, Shantakumar et al. conducted a study about the probability of BEC repeatability of COPD patients whether they have asthma or not and found that the Ri (Reliability index) value was 0.49 for COPD patients and 0.59 for those with asthma history [2]. Moreover, the study launched by Yoon et al. involved 618 patients with at least two BEC measurements. These patients were analyzed for longitudinal eosinophilic stability, and their asthma history was not excluded [6]. Also, Grenulich et al. conducted a multicenter, longitudinal study concerning the longitudinal stability of BEC when the history of asthma was not excluded [7]. The results were different and the specific data are indicated in Table 1.

Crudely, through comparison, more subjects with asthma history tend to be persistently higher than 150 cells/*μ*L (51.6% vs. 10.5). However, it is important to point out that the results were varied and unable to compare them further due to factors discussed later in this paper.

Asthma-COPD overlap (ACO) is a disease that has the features of both asthma and COPD and was first mentioned in the joint GOLD/GINA (Global Initiative for Asthma) in 2014 [8]. It may represent around 25% of COPD patients and around 20% of asthma patients [9]. Unified criteria of the diagnosis of ACO has been made by the sponsor of the Spanish COPD Guidelines (GesEPOC) and the Spanish Guidelines on the Management of Asthma (GEMA) among which elevated blood eosinophil count (≥300 cells/*μ*L) should be one of the diagnostics of ACO [10]. Yanagisawa et al. mentioned that an increase in the BEC levels might be a useful data point when exploring the possibility of ACO in patients who have already been diagnosed with COPD [11]. Few relevant studies have addressed the problem with inclusion criteria. Whether patients with ACO should be excluded remains controversial.

## 3. Definition of Stability

Currently, there are no standard criteria to define the stability of the BEC. Different studies have used various custom terms to define this, including the following:

1. “Predominantly eosinophilic” (PE): BEC ≥150 cells/*μ*L at each time or the presence of BEC <150 cells/*μ*L occurred only once; “rarely eosinophilic” (RE): BEC <150 cells/*μ*L or the presence of BEC ≥150 cells/*μ*L occurred only once; and “intermittently eosinophilic” (IE) none of the above-mentioned criteria were met [12].
2. “Concordance”: BEC persistently lower than or persistently higher than the absolute cutoff value 150 cells/*μ*L, 300 cells/*μ*L, or the percentage cutoff value 2%, 3%, and 4%; “discordance”: BEC varied between any two visits [3].
3. “Consistently high” (CH): both levels of BEC at baseline and 1-year follow-up were ≥250 cells/*μ*L; “consistently low” (CL): both levels were <250 cells/*μ*L'; “variably increasing” (VI): the baseline BEC was <250 cells/*μ*L and the BEC of 1-year follow-up was ≥250 cells/*μ*L; “variably decreasing” (VD): the baseline BEC was ≥250 cells/*μ*L and the BEC at 1-year follow-up was <250 cells/*μ*L [6].

Depending on the definition of various categories, the results appear to be different. It is difficult to compare these in parallel, so we cannot determine which is more reasonable. Thus, more scientific inquiries into BEC stability need to be further explored.

## 4. Data Analysis

**Figure 2:**
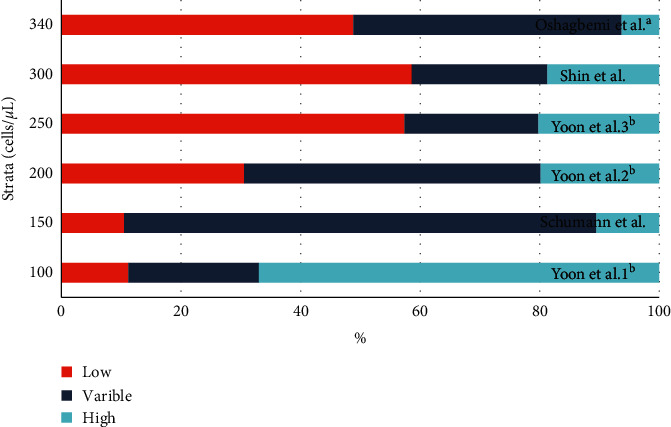
Distribution of BEC level with various strata from different studies. ^a^The data were recalculated based on the original data with longest observation time. ^b^The data were obtained from the same study with different strata levels.

The statistical analysis in each study was different, which might also influence the results. Several used the percentile of different categories as listed above. It is undoubted that various stratifications will influence the results (Figure 2).

**Figure 3:**
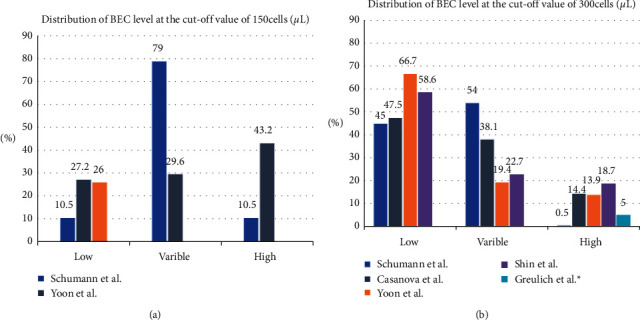
(a). Distribution of BEC level at the cutoff value of 150 cells/*μ*L.^*∗*^The variable and high group was absent in the study launched by Greulich et al. (b) Distribution of BEC level at the cutoff value of 300 cells/*μ*L.^*∗*^The variable and low group was absent in the study launched by Greulich et al.

However, even with the same strata the results are still quite different (Figure 3). Although the percentile of BEC is easily accessible, it remains a latent problem that some patients' measurements could be several months or years apart and others could be days or weeks apart. However, there may have been circumstances where BEC fluctuated between measurements even though the final total range remains unchanged.

**Figure 4:**
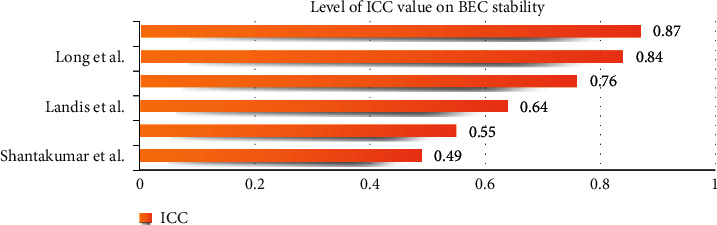
Level of ICC value on BEC stability from different studies. ICC, intraclass correlation coefficient. ICC value: excellent (>0.75), fair to good (0.40–0.75), and poor (<0.40).

An intraclass correlation coefficient (ICC) was used to estimate measurement repeatability [13]. Several studies chose ICC to evaluate the stability of BEC [2, 6, 14–17]. Many of these studies produced reassuring results of fair to good (0.40–0.75) or excellent (>0.75), which indicates that BEC stability was good (Figure 4).

Strictly speaking, while using the ICC, a different descriptive statistical model should have been chosen first [18]. It is regrettable that the information was not communicated in the studies. A few other statistical tools were also used while evaluating stability. For example, Fleiss's kappa—a descriptive statistical model—was first used by Nishimura et al., yielding a result of 0.464, which shows a moderate level of BEC stability [17]. There are no available data to compare the same statistical model to evaluate the credibility of each.

## 5. Thresholds

In addition, the thresholds matter. Different studies set different thresholds and cutoff values within their own groups as subscribed before [19]. Thus, the capacity of patients was categorized according to different strata, which will surely lead to variability in the results. The GOLD guidelines set the cutoff value at <100 cells/*μ*L and >300 cells/*μ*L. Nevertheless, it reaffirms that these thresholds should be regarded as estimates rather than precise cutoff values. As is shown above, the data indicates a large number of patients were located on intervals based on the above strata, which means ICS usage tends to be vague. According to Yoon et al., the ideal cutoff seems to depend on the correlation between the baseline and follow-up BEC [6]. Accordingly, it is hopeful that with the deeper exploration of BEC in the future, these thresholds will be much more precise.

## 6. Oral Corticosteroid

Whether or not to exclude patients who were using oral corticosteroid (OCS) remains controversial. Some studies excluded patients who had used OCS within the previous thirty days or longer in case the influence of BEC affected the results [15, 20]. Sivapalan et al. conducted a multicenter, randomized, controlled, open-label, noninferiority trial to test eosinophil-guided corticosteroid therapy with 318 COPD patients and found a reduction of BEC after treatment with systemic corticosteroid therapy [21]. However, Shine et al. found the use of systemic corticosteroid did not change the BEC among groups of COPD patients during the median follow-up of six years [4]. Meanwhile, Schumann et al. found no correlation between baseline BEC level and whether the systemic corticosteroid was used or not while studying the stability of EOS in COPD patients [3]. Landis et al. stated that oral prednisone was associated with a reduction of BEC that returned to baseline after the wash-out period, suggesting that OCS should be excluded [15]. According to the above, aside from the various points mentioned in this paper, the factors leading to this difference in results are mainly the duration of systemic corticosteroid use. Current studies did not find a correlation between BEC stability and ICS use in COPD patients [5, 22]. We propose a wash-out period for patients who currently use or have used OCS in the past in case it influences the BEC. The GOLD guidelines did not recommend the use of OCS in the stable phase unless they were in different grades of exacerbation, which is beyond the scope of our study.

## 7. Smoking

Smoking appears to present an uncertain risk factor for COPD patients. Salvi et al. made a comparison between nonsmoking COPD (NS-COPD) and smoking COPD (S-COPD) patients with two-year follow-up. They found NS-COPD subjects had 1.4 times more sputum EOS than S-COPD subjects, but there was no difference in BEC counts between S-COPD and NS-COPD subjects [23]. However, the two groups have some variance in age, BMI, and sex at baseline, which is statistically significant. In addition, Hartl et al. measured the BEC in a random sample of 11,042 subjects recruited from the general population and found that a current smoking habit was significantly associated with a higher BEC, but former smoking as well as high cumulative smoking exposure (≥20 pack years) was not (1.11 [0.96–1.27]) [24]. However, Oshagbemi et al. did not find any difference in BEC stability between current smokers and nonsmokers among COPD patients [20]. Casanova et al. used two large cohort studies comprising 1,746 smokers to investigate the stability of BEC with remarkable results of the stability of BEC [5]. However, these results can only represent the portion of COPD patients who were smokers rather than the overall group. Thus, smoking remains an uncertain factor, and the underlying mechanisms need to be explored in detail.

## 8. Others

Some other risk factors might also influence the results. First, racial differences might play an important role. The above studies covered several countries and found that race might influence BEC stability. Shantakumar et al. found that Maori/Pacific COPD patients may have a higher BEC because of genetic predisposition or lifestyle factors [2]. This is the first time a study has proposed that BEC varies according to ethnic differences in COPD patients. A large prospective cohort with ethnicity related studies is urgently needed. In addition, the results might correlate with study capacity regardless of other factors. Second, and as mentioned above, the duration of follow-up in each study differs significantly. According to Schumann et al., the stability of BEC lowered by 23% within eighteen months [3]. Aside from this, when we consider the circadian rhythm, BEC is highest between 6  hours and 12  hours [3].

## 9. Mechanisms

As the GOLD guidelines depict, the mechanism of BEC in COPD patients remains unclear. To further investigate whether the BEC is stable and to further discover the latent capacity of the BEC as a surrogate biomarker of COPD, the mechanisms and genetic impact on blood EOS levels should be further explored. Interleukin-5 (IL-5), GATA binding protein 3 (GATA3), and eotaxin (CCL11, CCL24) are reported as crucial cytokines and/or transcription factors in eosinophilic COPD [25, 26]. These findings can provide us a lead in exploring the mechanism of eosinophilia in COPD. Genetically, George et al. used samples of bronchial epithelial brush and an RNA-Seq from COPD patients, but they failed to find any change in gene expression between subjects categorized as eosinophilic versus noneosinophilic as determined by BEC of >200 cells/*μ*L [27].

## 10. Conclusion

BEC is a good biomarker in predicting COPD outcomes. However, the stability of BEC still remains controversial due to study outcomes with varying results. After analyzing those studies, we argue that more real-world cohort studies with longitudinal tracing times and more frequent BEC measurements of native COPD patients are urgently needed. Meanwhile, stricter inclusion criteria should be considered, such as age, smoking status, ethnicity, circadian rhythm, and the use of OCS. When analyzing the data, the ICC can be used rather than the percentile. Clinically, when prescribing ICS to a COPD patient, overlapping measurements of BEC are needed during stable phases. In addition, an evaluation system should be constructed to provide different levels of recommendation based on figures such as sputum EOS counts, frequency of acute exacerbations, and so forth. With the mechanisms of BEC in COPD being further explored, it is hoped that BEC will better guide the clinical management of COPD patients in the future.

## Figures and Tables

**Table 1:** Results of BEC level with or without asthma.

	With asthma				Without asthma			
	Greulich et al.	Casanova et al. 1^a^	Yoon et al.	Mean	Schumann et al.	Shin et al.	Casanova et al. 2^b^	Mean
Lower than 150 cells/*μ*L (%)	26	6	27.2	19.7	10.5	N	N	10.5
Higher than 150 cells/*μ*L (%)	*N* ^c^	60	43.2	51.6	10.5	*N*	*N*	10.5
Lower than 300 cells/*μ*L (%)	N	40.3	66.7	53.5	45	58.6	57.5	53.7
Higher than 300 cells/*μ*L (%)	5	15.8	13.9	11.6	0.5	18.7	12.3	10.5
Measure frequencies	3	3	2	2.7	9.8	2	3	4.9
Observation time (month)	18	26	12	18.7	90	72	48	70
Number of observers (*n*)	165	424	618	402.3	210	299	308	272.3

^a^One of the cohorts with two strata value used in the research. ^b^Another cohort in the research. ^c^*N*: results were not available in the research.

## Data Availability

No data were used to support this study.
